# Revealing associations between spatial time series trends of COVID-19 incidence and human mobility: an analysis of bidirectionality and spatiotemporal heterogeneity

**DOI:** 10.1186/s12942-023-00357-0

**Published:** 2023-11-27

**Authors:** Hoeyun Kwon, Caglar Koylu

**Affiliations:** Department of Geographical and Sustainability Sciences, University of Iowa, Iowa City, IA USA

**Keywords:** COVID-19, Human mobility, Granger causality, Dynamic time warping, Spatial time series analysis

## Abstract

**Background:**

Using human mobility as a proxy for social interaction, previous studies revealed bidirectional associations between COVID-19 incidence and human mobility. For example, while an increase in COVID-19 cases may affect mobility to decrease due to lockdowns or fear, conversely, an increase in mobility can potentially amplify social interactions, thereby contributing to an upsurge in COVID-19 cases. Nevertheless, these bidirectional relationships exhibit variations in their nature, evolve over time, and lack generalizability across different geographical contexts. Consequently, a systematic approach is required to detect functional, spatial, and temporal variations within the intricate relationship between disease incidence and mobility.

**Methods:**

We introduce a spatial time series workflow to investigate the bidirectional associations between human mobility and disease incidence, examining how these associations differ across geographic space and throughout different waves of a pandemic. By utilizing daily COVID-19 cases and mobility flows at the county level during three pandemic waves in the US, we conduct bidirectional Granger causality tests for each county and wave. Furthermore, we employ dynamic time warping to quantify the similarity between the trends of disease incidence and mobility, enabling us to map the spatial distribution of trends that are either similar or dissimilar.

**Results:**

Our analysis reveals significant bidirectional associations between COVID-19 incidence and mobility, and we develop a typology to explain the variations in these associations across waves and counties. Overall, COVID-19 incidence exerts a greater influence on mobility than vice versa, but the correlation between the two variables exhibits a stronger connection during the initial wave and weakens over time. Additionally, the relationship between COVID-19 incidence and mobility undergoes changes in direction and significance for certain counties across different waves. These shifts can be attributed to alterations in disease control measures and the presence of evolving confounding factors that differ both spatially and temporally.

**Conclusions:**

This study provides insights into the spatial and temporal dynamics of the relationship between COVID-19 incidence and human mobility across different waves. Understanding these variations is crucial for informing the development of more targeted and effective healthcare policies and interventions, particularly at the city or county level where such policies must be implemented. Although we study the association between mobility and COVID-19 incidence, our workflow can be applied to investigate the associations between the time series trends of various infectious diseases and relevant contributing factors, which play a role in disease transmission.

## Introduction

As COVID-19 transmits through exposure to respiratory fluids from infected people, non-pharmaceutical interventions (NPIs) that limit human social interactions, such as travel restrictions and nonessential business closures, were implemented to delay the spread of COVID-19. To ensure the effectiveness of the NPIs, prior studies examined the relationship between human social interactions and COVID-19 transmission. As a proxy to measure human social interactions, human mobility data captured from anonymized mobile phone location records have been widely used in efforts combatting COVID-19 [1–3]. Previous studies found positive correlations between human mobility and COVID-19 incidence [4, 5]. For example, as there were a greater number of travels within and into a city, COVID-19 incidence of that city increased, especially in the early phase of the outbreak [6]. On the other hand, as COVID-19 cases increased faster, daily travel distances were reduced more quickly [7]. Furthermore, the relationship between human mobility and disease incidence was found to be non-stationary in time [1, 8, 9] and across geographic space including small scales such as census tracts [10, 11]. Identifying these spatial and temporal variations is important because they help reveal confounding factors and processes that alter the disease dynamics. For instance, a severe pandemic situation may intensify the practice of social distancing, which could directly reduce human mobility, while risk perception and the fear of infection caused by such severity could indirectly decrease mobility by making individuals stay more at home [12, 13]. While the existing studies have revealed the spatially and temporally varying relationship between disease incidence and human mobility, only a few studies have focused on the fact that both disease incidence and mobility may influence each other [13, 14]. Also, such bidirectional relationships are still not generalizable across all geographies and may also change over time. Ultimately, there is a lack of a systematic approach to identify the complex bidirectional associations among disease incidence and mobility.

In this study, we introduce a spatial time series analysis workflow to investigate the complex bidirectional associations between human mobility and disease incidence, examining how these associations evolve over time (across different waves) and vary across diverse geographical regions. Using different phases of the COVID-19 pandemic as our case study, we construct daily time series data for COVID-19 cases derived from U.S. COVID Risk and Vaccine Tracker [15] and human mobility flows at the county level in the U.S. derived from anonymized and aggregated mobile phone location data from SafeGraph [16]. To capture potential social interactions resulting from people's movements, we aggregate mobility flows within and into each county, encompassing not only intra-county movements but also flows from other counties across the country [9]. By employing two-way Granger causality tests, we examine the associations between the time series trends of COVID-19 cases and mobility flows, capturing bidirectional relationships between these variables. We develop a typology to identify and explain how the relationship between disease incidence and mobility changes in terms of significance, directionality, and spatiotemporal variability across different waves for each county of the U.S. Furthermore, we utilize dynamic time warping to quantify the similarity between the trends of disease incidence and mobility, enabling us to map the geographical variations in these trends.

## Related work

With the outbreak of COVID-19, NPIs such as lockdowns and stay-at-home orders have been implemented in the U.S. since mid-March 2020, which resulted in a substantial decrease in human mobility. Here, human mobility is a proxy for social interactions and spatial colocation of individuals that allow the transmission of the disease. Specifically, a significant decrease in travel time [17] and a long-term reduction of long-distance mobility were caused by NPIs, and specifically, lockdowns [5]. Findings of previous studies suggested that lockdowns limited human mobility and thus helped flatten the curve of COVID-19. Studies conducted at the global or national scale revealed that human mobility and COVID-19 incidence were positively correlated [6, 18]. However, it is challenging to elucidate their correlations since COVID-19 cases and human mobility affect each other simultaneously [13]. Moreover, the interactions between COVID-19 incidence and human mobility may vary by geography and time. In addition to revealing a universal correlation between mobility and COVID-19 incidence at the global or national level, several studies analyzed human mobility flows at finer scales (e.g., census block, county, state) using mobile phone location data and found local variations in their association [7, 10, 11]. At the state level, Gao et al. [7] examined the changes in human mobility according to a stay-at-home order and found that the relationship between mobility and COVID-19 incidence varied across different states. Similarly, but at a finer scale, a case study analyzing two distinct counties revealed that the associations between the spread of COVID-19 and human mobility varied substantially across areas even within the same county [10].

Temporal variations in the association between COVID-19 incidence and human mobility have also been examined. Katragadda et al. [19] showed that both local population mobility and visitors’ mobility elevate the COVID-19 transmission risk, and the way how these two types of mobility may affect the disease transmission varies across states and phases of the COVID-19 pandemic. This study revealed that the influence of local mobility on disease transmission was predominant in the early stage of the pandemic and became weaker over time. Xiong et al. [9] also revealed that the degree of correlation between COVID-19 cases and human mobility varied over time at the county level in the Xiong et al. [9] grouped the counties into two categories—whether lock-downed or reopened, but did not focus on spatial heterogeneities in the time series correlation between disease incidence and mobility. However, understanding both spatial and temporal heterogeneities simultaneously is necessary since time series correlation may vary geographically, even at small scales. Jewell et al. [1] demonstrated time-varying relationships between human mobility and COVID-19 incidence across different regions. This study provides evidence of spatial and temporal variations in the relationship between mobility and COVID-19 incidence, however, it is focused on one direction of the relationship (i.e., how mobility affects COVID-19 incidence). There is lack of a systematic approach to assess those heterogeneities across different directions of the relationship between disease incidence and mobility.

Many factors, such as risk perception, fear of infection, trust in the healthcare system, main occupation, and means of transport, also affect COVID-19 incidence directly or indirectly [12, 13, 17]. For example, Borkowski et al. [17] uncovered that larger families decreased their mobility more than smaller families. As they also found that people decreased traveling as they were more afraid of getting infected, bigger household sizes may increase the risk of infection and make people reduce mobility. Prior studies also found that the local variations in COVID-19 incidence were significantly affected by population distribution, age structures, and racial heterogeneity [10, 11]. These previous studies suggest that the COVID-19 incidence has spatial and temporal variations depending on not only human mobility flows but also complex sociodemographic processes and factors, especially in highly populated metropolitan areas. Although it is perhaps impossible to reveal the causal factors that alter the relationship between mobility and disease, in this article, our goal is to capture the non-stationary relationships that could help us develop hypotheses for explaining the complex relationship between social interactions and disease dynamics.

## Methods

**Fig. 1:**
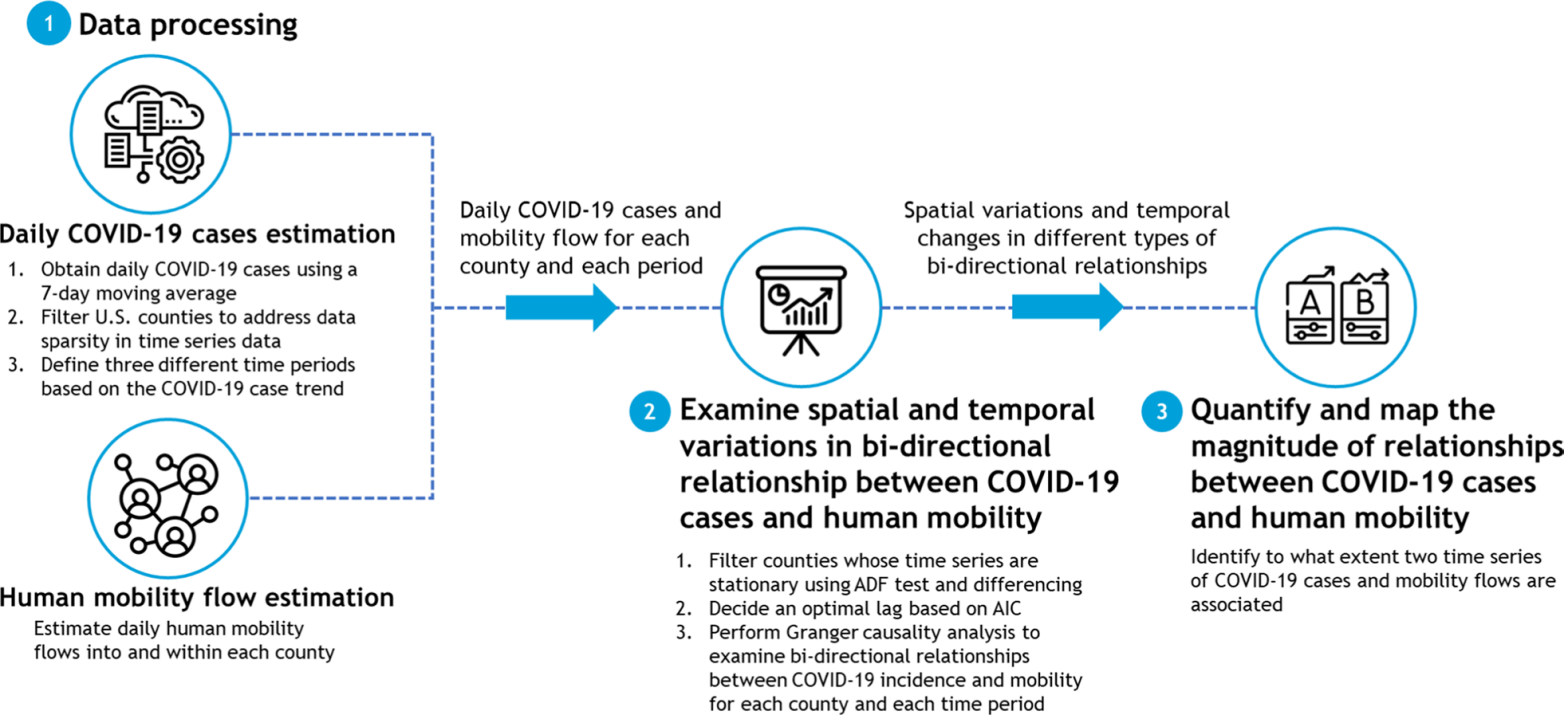
Spatial time series workflow

We introduce a spatial time series analysis workflow to examine the bidirectional associations between human mobility and disease incidence and how such associations may vary over time and across different geographies at the county level in the U.S. Our workflow consists of four major steps (Fig. 1). We first process the COVID-19 data to get daily new COVID-19 cases trend at the county level for three waves of the pandemic. We also estimate daily human mobility flows into and within each county using anonymous mobile phone location data. Then, we perform two-way Granger causality tests for each county to determine if there are any significant bidirectional associations between the time series trends of daily COVID-19 cases and mobility flows. Finally, we use the dynamic time warping method to identify the form of the relationship, either positive or negative association, and measure the magnitude of such association by standardizing the DTW measure. Although we study the association between human mobility and disease incidence using COVID-19 as a case study, our workflow is generic and can be applied to study time series associations between two different spatiotemporal phenomena.

### Case study and data

We use COVID-19 cases and human mobility flows of each county in the contiguous U.S. We collect the COVID-19 case data from COVID Risk and Vaccine Tracker [15], which is open to public through API. They provide many metrics, including daily new COVID-19 cases for every county in the U.S. We then smooth daily number of new COVID-19 cases by applying 7-day moving average. For human mobility data, we use the data derived from anonymous and aggregated SafeGraph mobile phone location data [16]. We estimate human mobility flows between every pair of counties by extrapolating the sample SafeGraph data, which we describe more details in the next section. The time periods used in this study are the first wave is from April 1st, 2020, to May 31st, 2020; the second wave is from June 1st, 2020, to August 31st, 2020; and the third wave is from November 1st, 2020, to January 31st, 2021. These time periods are defined while considering seasonality, covering the peaks of the COVID-19 case trend, and keeping the length of each period similar. For the spatial time series analysis, we use counties in metropolitan statistical areas. We also filter out counties whose average number of COVID-19 cases during our study period is less than the median value of the cases of all counties. This is to avoid the problems due to data sparsity since spatial time series analysis requires data to be disaggregated by both spatially and temporally.

### Estimating human mobility flows between counties

We obtain the mobility data from SafeGraph [16]. Data consist of the number of trips between every pair of census block groups derived from anonymous mobile phone locations, and each visit is a flow from one block group to another or an internal trip within a block group. The sample corresponds to approximately 10% of all mobile phone users. To estimate the population mobility between an origin block group O and a destination group D, we apply the following equation [20]:

$$Mobility \left( O, D\right) = Devices \left( O, D \right)\times Population\left( O \right)/Devices \left( O \right)$$

Mobility from origin block group O to destination block group D is estimated by multiplying the number of devices that moved from O to D with the population of O divided by the number of devices in O. This formula provides a bulk estimate of the population mobility based on the assumption that populations who are not captured in the sample data had the same mobility behaviors as the captured populations do. We then aggregate block group to block group population flows into to county-to-county flows. Once we obtain mobility flows between every pair of counties, we compute inflow and within-flow measures for each county.

$$Inflow_i = \mathop{\sum} \limits_{j \ne i}^n flow_{ji} \quad Withinflow_i = flow_{ii}$$

where inflow is the sum of all incoming flows into a county, and within-flow is the sum of mobility flows within a county (i.e., flows whose origin and destination county are the same). We are able to compute the flows that happen within a county since the original flow data is acquired at the block group level. $flow_{ji}$ denotes the number of visitors from origin county $i$ to destination county $j$, and $n$ denotes the number of origin counties.

In this study, we use the sum of inflows and within-flows in a county as a mobility measure. These two metrics capture distinct aspects of human mobility. Inflow measures the movement of people from other counties into the target county, while within-county flow tracks the movement of individuals already residing within the county. To understand the full scope of potential disease spread, it's essential to consider both these types of movement. Inflows can introduce the virus and its new variants into the county, while within-county movement can facilitate the spread within the local population. The nature of inflow and within flow movements is likely to be different. Inflow movements are often influenced by factors like commuting for work, tourism, or regional events, which can vary significantly based on geographic, economic, or seasonal factors. On the other hand, within flows are typically related to not only commuting but also daily life activities such as shopping, school, and social gatherings. Therefore, we include both inflows and within-flows to avoid bias in our understanding of the relationship between mobility flows and disease incidence.

Finally, we apply a 7-day moving average smoothing to our daily mobility measure (sum of inflows and within-flows) for each county to reduce daily fluctuations and sampling issues. This approach aligns our mobility data processing with the 7-day average smoothing used for COVID-19 case counts, ensuring consistency in our analysis.

### Examining bidirectional associations between two time series across space and time

To investigate the associations between two time series trends, we use the Granger causality test. Granger causality tells us whether the past values of one variable could be used to forecast or explain the changes in another variable in the time series. The statistical test for Granger causality evaluates whether variable A can improve the prediction of another variable B in the time series. The test also accounts for the case that a single variable can predict its own trend. Thus, the test evaluates whether information from both A and B predicts B better than B predicting its own trend. Granger causality shows whether two variables are associated in their time series and is different from cause-and-effect relationships. The Granger causality test is useful in our study since it provides statistical evidence of whether COVID-19 cases and human mobility have any significant relationships. We should note that unlike actual causality, which implies a direct cause-effect relationship, Granger causality in time series analysis merely suggests that one variable can be used to forecast another, without implying a true causal link.

#### Testing the stationarity of data and identifying the optimal lag

There are multiple steps for running the Granger causality tests for spatial time series data. First, we examine if time series data are stationary to eliminate the possibility of temporal autocorrelation that would skew the test results. The Granger causality test assumes that two time series to be analyzed are stationary because nonstationary data may produce misleading and spurious correlations [21]. Stationary time series data should have constant mean and variance without seasonal trends. We use the Augmented Dickey–Fuller (ADF) test [22] which is one of the most popular statistical methods to test time series stationarity. If the ADF test result shows that the data is not stationary, data transformation such as differencing can be applied. For example, one can use the differences between consecutive values (i.e., change of the values from one time point to the next), which help stabilize the mean and eliminate seasonal trends. The stationarity of transformed time series data also needs to be tested to perform the Granger causality test. Second, we determine the lag which is a time gap between two time series to be incorporated into the analysis. We decide the optimal lag based on the Akaike information criterion (AIC) [23] which is a commonly used estimator of the optimal lag length in time series analysis. AIC evaluates how well the model explains the data, and generally, AIC score is lower as a model fits better.

#### Identifying significant time series correlations using the Granger causality test

Using the stationary time series data and the optimal lag determined in the previous steps, we perform the Granger causality test. The model with two stationary time series $X_t$ and $Y_t$ can be illustrated mathematically with the following equation [24]:

$$X_t = \mathop \sum \limits_{j = 1}^m a_j X_{t - j} + \mathop \sum \limits_{j = 1}^m b_j Y_{t - j} + \varepsilon_t$$

where $m$ is the maximum lag, $a$ and $b$ are coefficients (i.e., the effect of lag $j$), and $\varepsilon$ is noise. In this study, we test two null hypotheses for each pair of spatial time series data: (1) COVID-19 cases do not Granger-cause mobility flows, and (2) Mobility flows do not Granger-cause COVID-19 cases. We reject the null hypotheses if a *p*-value for a Chi-Square test is less than 0.05. We perform the test in both directions since the results of the two tests are independent. Therefore, by testing these two hypotheses, we define four different cases in bidirectional relationships: (1) COVID-19 incidence Granger-causes human mobility, (2) human mobility Granger-causes COVID-19 incidence, (3) Both COVID-19 incidence and mobility Granger-causes each other simultaneously, and (4) There is no significant relationship between the trends of mobility and COVID-19. Furthermore, to examine spatial and temporal variations, these two hypotheses are tested for each county and each of three waves separately.

### Measuring similarities of two time series by county

Although the Granger causality test examines if each county has a statistically significant correlation between COVID-19 infection and human mobility, it does not tell us whether two time-series trends of human mobility and COVID-19 cases have a positive or negative correlation. We cannot quantify the magnitude of their correlation using the Granger causality test, either. Therefore, we use the dynamic time warping (DTW) method to measure the similarity between COVID-19 cases and human mobility flows and map the spatial distribution of the similar and dissimilar time series trends [25]. DTW compares two or more time series by handling different lengths, noise, shifts, and amplitude changes [26, 27]. DTW computes a distance measure between two time series, which becomes smaller as those two series have more similar trends. Before computing DTW distances, we normalize all series using the min–max approach and rescale them to have a fixed range of [0, 1]. Normalization allows us to compare DTW values of different counties (i.e., spatial time series to each other by standardizing different ranges of values and units in different time series data. Thus, we calculate a DTW distance between the standardized human mobility and COVID-19 time series for each county to investigate the similarity between the two variables.

## Results

### Time series association between COVID-19 incidence and human mobility

Before conducting time series analysis, we first filtered the counties among the 588 metropolitan statistical areas that had an average number of COVID-19 cases greater than the median number of cases of all counties during our study period. By differencing the data and conducting the ADF test, we identified 425 counties that had stationary time series for both COVID-19 cases and human mobility. Then, to find an optimal lag length for the Granger causality test, we computed AIC for each county and then calculated the mean of AIC values of all counties for each wave. As a result, in all three waves, the curve of mean AIC values of counties flattens around lags of seven and eight days, and then AIC maintains similar values as the lag length becomes longer. We chose to use a 7-day lag due not only because it has the lowest AIC values in waves 2 and 3, but also there is evidence from the previous studies of the earlier waves that found a 7-day lag between the contact with an infected person and the manifestation of clinical symptoms [9].

**Fig. 2:**
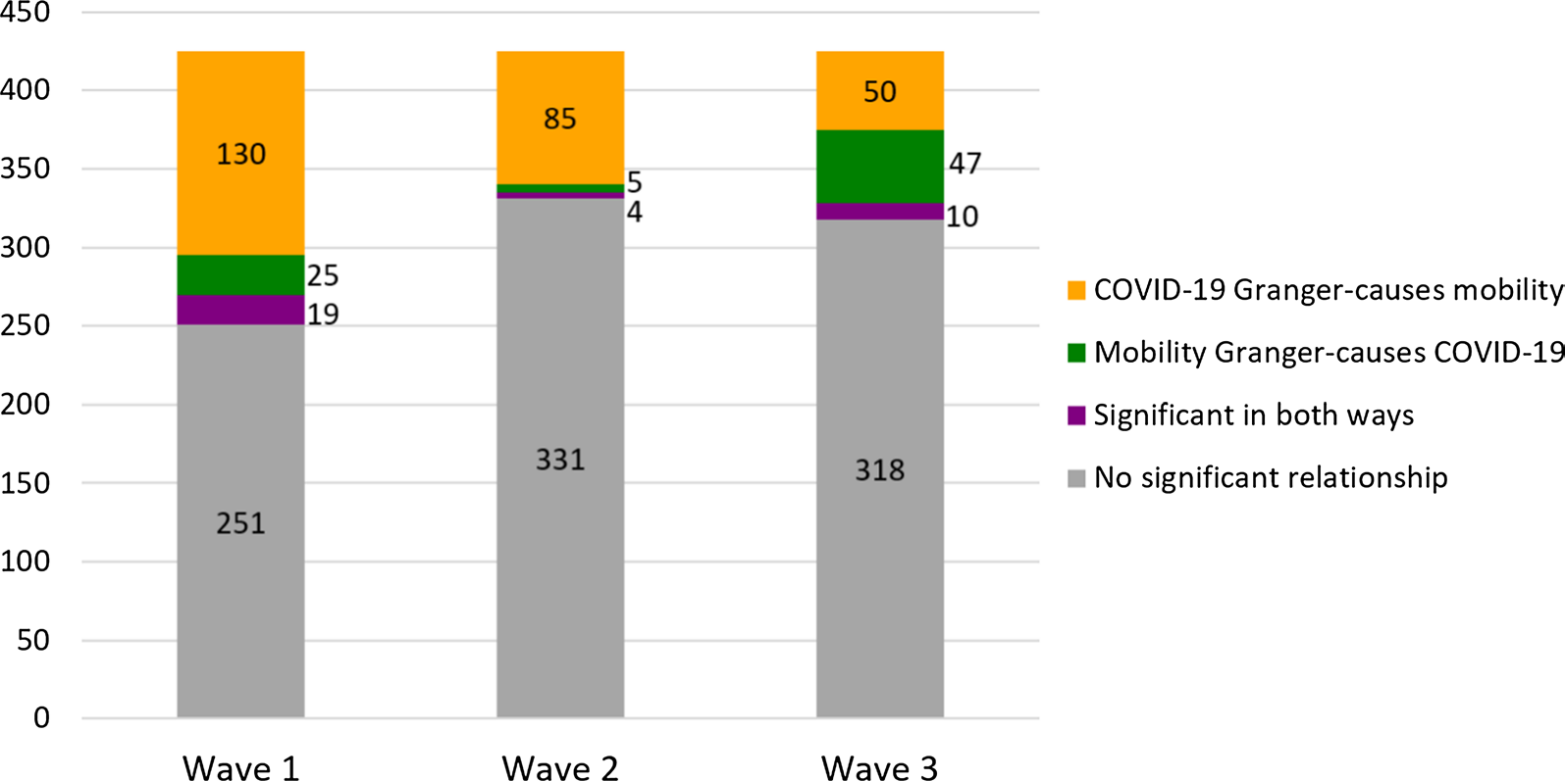
The number of counties with statistically significant relationships by directions

Next, we performed the Granger causality analysis for each of the three waves to identify the significant time series associations between COVID-19 cases and human mobility flows. Figure 2 shows the number of statistically significant counties for each of four possible cases in bidirectional relationships: (1) COVID-19 incidence Granger-causes human mobility, (2) human mobility Granger-causes COVID-19 incidence, (3) significant in both ways, and (4) no significant relationship. The results in Fig. 2 reveal some interesting findings regarding the temporal variations in those bidirectional relationships. First, more than half of the counties do not have any statistically significant relationship between COVID-19 incidence and mobility across all periods. Second, among the counties with significant relationships, overall, it is more common for COVID-19 cases to have a stronger effect on human mobility than human mobility does on COVID-19 incidence across different periods mainly because of NPIs. Third, the number of counties where COVID-19 cases Granger-cause mobility flows is 130, 85, and 50 in waves 1, 2, and 3, respectively. The number keeps decreasing over the three waves. This supports the fact that human mobility was most strictly restricted during the earlier periods of the pandemic due to the enforced stay-at-home orders [28] and the fear of infection was also the highest in this early phase of the pandemic [12]. On the other hand, mobility has a significant influence on COVID-19 cases in only a few counties, which shows that mobility flows do not Granger-cause COVID-19 cases as much as the effect of COVID-19 on mobility. However, it is notable that the number of counties where mobility Granger-causes COVID-19 cases increase sharply in wave 3. It implies that the increase in mobility due to the reopening policies and the availability of COVID-19 vaccines in late 2020 results in having more influence on COVID-19 cases.

**Fig. 3:**
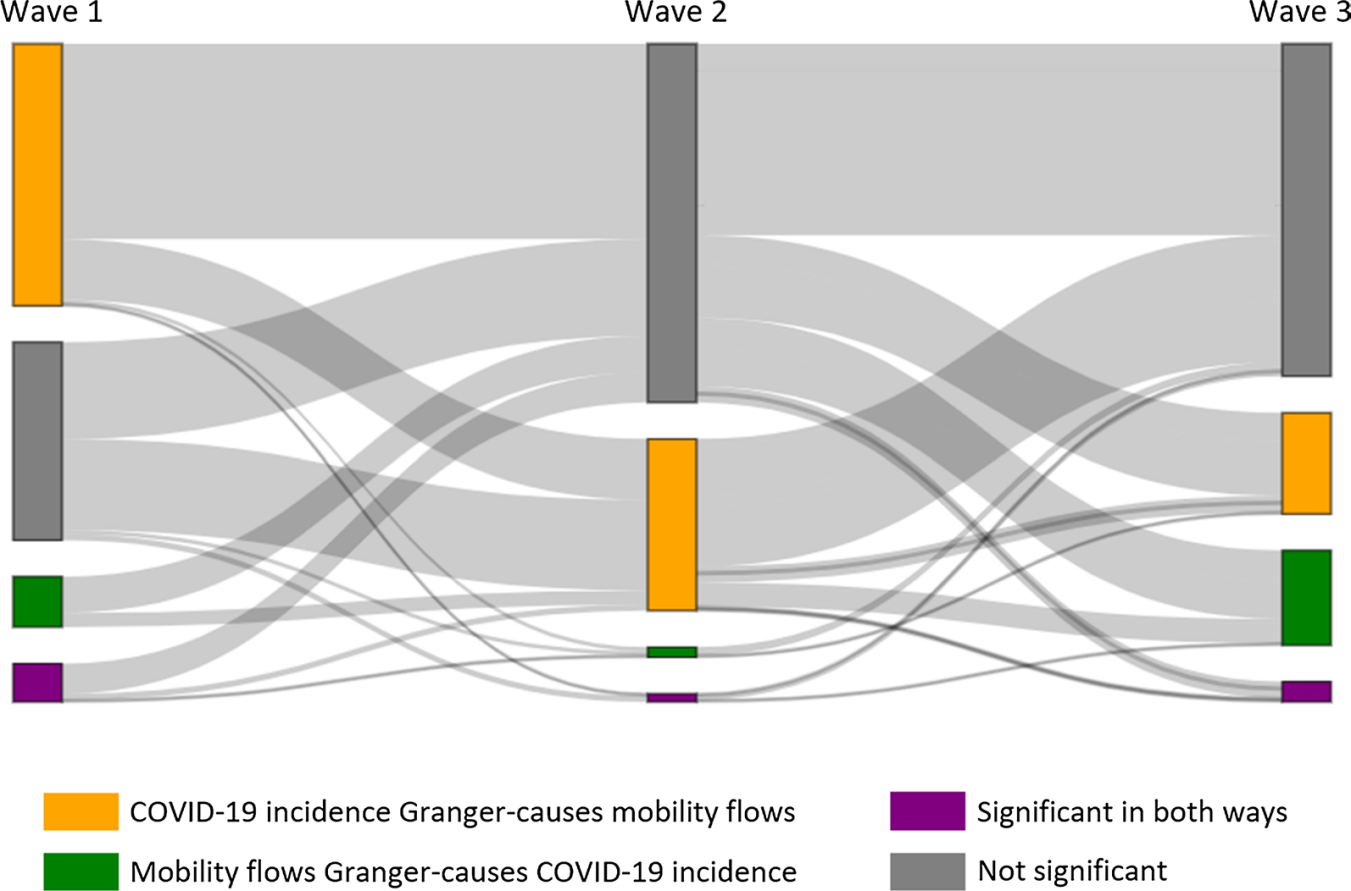
The number of counties that changed or remained stable in terms of the direction and significance of the association between disease incidence and mobility

We further disaggregate this time-series trend in Fig. 2 and investigate more detailed temporal patterns on how relationships between COVID-19 cases and human mobility changed or remained stable. Figure 3 shows how each of four possible cases in bidirectional relationships evolves throughout three different waves of the pandemic. In Fig. 3, the width of grey flow lines indicates the number of counties. Note that this Sankey diagram only includes counties with at least one significant relationship over three waves.

The most frequent pattern of change (i.e., the widest flow line) is that COVID-19 incidence Granger-causes mobility in wave 1 without any significant relationship in waves 2 and 3. This result supports the previous findings that the correlation between COVID-19 cases and mobility was the strongest during the initial lockdown and became weaker since other factors resulted in complex disease dynamics as time passed [8]. Another notable pattern is that approximately half of the counties without any significant relationships in wave 1 changed to have a significant relationship that COVID-19 incidence Granger-causes mobility in wave 2, and the relationship of more than half of those counties again became insignificant in wave 3. Such information is hidden when aggregated as in Fig. 2, although we can still find the general trend of decreasing number of counties where COVID-19 cases Granger-cause mobility from Fig. 2.

**Fig. 4:**
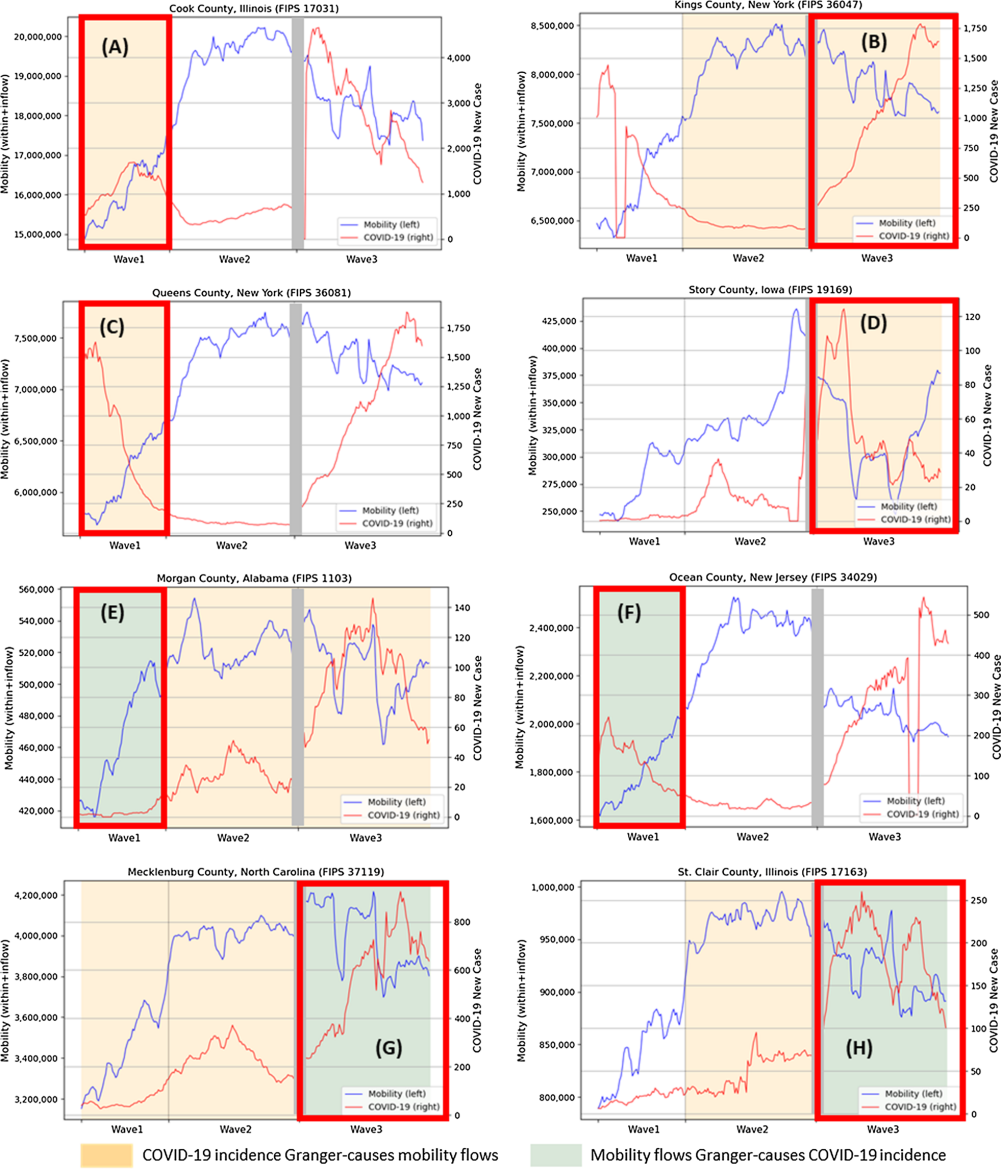
Eight possible scenarios in the bidirectional relationship between COVID-19 incidence and mobility flows. Each of **A**–**H** in a red box indicates each scenario

Although Granger causality helps identify the direction and significance of the association between two time series trends, these associations can vary in form such as positive and negative associations. Consequently, each of the four associations in Fig. 3 could have two distinct forms, which makes a total of eight possible scenarios. Figure 4 illustrates the time series trends of these eight distinct scenarios drawn from our county-level time series analysis results. When COVID-19 incidence Granger-causes human mobility with a negative association, there are two possible scenarios. In Kings County, NYC, the increasing trend of COVID-19 incidence Granger-causes and precedes the decreasing trend of mobility in (Fig. 4b). Second, the decreasing COVID-19 incidence Granger-causes and precedes the increasing mobility trend in Queens County, NYC (Fig. 4c). The presence of these two distinct forms of associations can be attributed to specific behavioral patterns observed during the pandemic. When the disease incidence is on the rise, people tend to exhibit increased caution and reduce their mobility, leading to a subsequent decline in mobility trends (as observed in Kings County, NYC). Conversely, when the pandemic situation is less severe and disease incidence is declining, individuals tend to become more willing to travel, resulting in a subsequent increase in mobility trends (as observed in Queens County, NYC). These patterns highlight the relationship between the severity of the pandemic and individuals' willingness to travel, ultimately influencing the direction of the association between COVID-19 incidence and mobility. The association can also be positive in the form of increasing or decreasing trends. Figure 4a illustrates a positive association such that both COVID-19 cases and mobility flows are increasing, while Fig. 4d illustrates that both trends are decreasing. In both cases (Fig. 4a, d), the trend of COVID-19 Granger-causes and precedes that of mobility flows. This implies that human mobility flows are affected by many factors other than the pandemic severity, such as better mask-wearing and social distancing policies, which is also discussed in the previous study [1].

Likewise, when mobility flows Granger-cause and precede COVID-19 incidence, the association can be both positive and negative. The increasing COVID-19 cases can be followed by the increasing mobility flows (Fig. 4e) since increased mobility can be a proxy of more social interactions and a higher risk of infections. On the contrary, the decreasing trend of mobility can Granger-cause the decreasing COVID-19 incidence (Fig. 4h). However, if there are negative associations, it is also possible that the trend of COVID-19 cases is increasing although mobility flows are decreasing (Fig. 4g) or vice versa (Fig. 4f), which implies confounding factors affecting the COVID-19 incidence trend.

We further investigated the distribution of the counties with significant Granger-causality across eight types of scenarios. To do so, for each county and for each wave, we performed the analysis of (1) Pearson correlation to examine if two trends of COVID-19 and mobility have a positive or negative association and (2) a simple linear regression to decide if each of COVID-19 and mobility trends is increasing or decreasing. As a result, we found that when COVID-19 trend Granger-causes mobility trend, they are more likely to have positive correlation where both trends are increasing in wave 1 and wave 2. In wave 3, however, the most common scenario is that those two trends have a negative correlation where COVID-19 cases are increasing, and mobility flows are decreasing. On the other hand, when mobility trend Granger-causes COVID-19 trend, those two trends are more likely to have negative associations in waves 1 and 3, but positive associations are more common in wave 2. More detailed results can be found in the Appendix.

### Spatial and temporal variations of COVID-19 incidence and mobility relationship

**Fig. 5:**
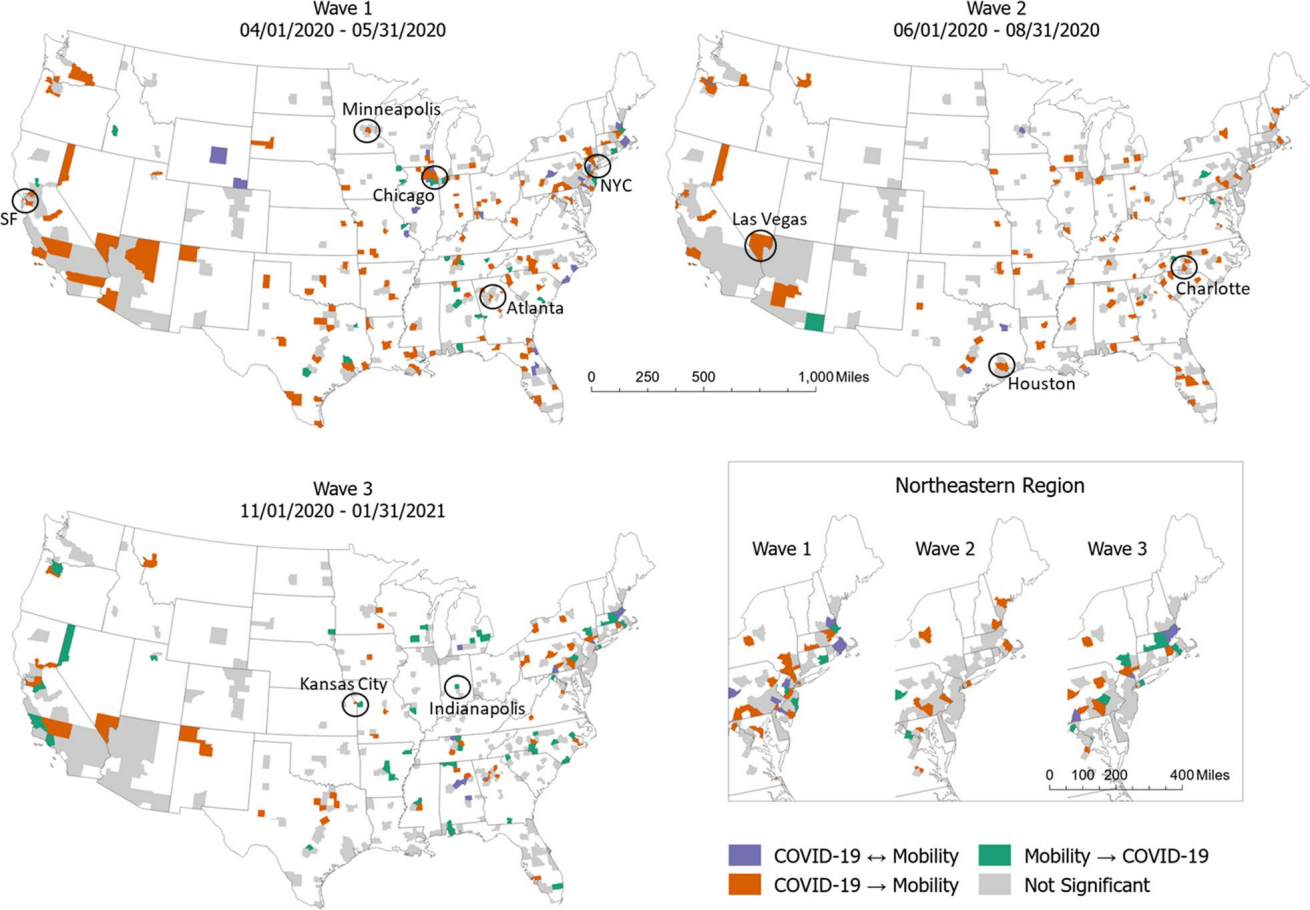
Spatial and temporal variations of COVID-19 and mobility relationship. (‘COVID-19 ↔ Mobility’, ‘COVID-19 → Mobility’, ‘Mobility → COVID-19’, and ‘Not Significant’ indicates ‘COVID-19 and Mobility simultaneously’ Granger-cause each other’, ‘COVID-19 Granger-causes mobility’, ‘Mobility Granger-causes COVID-19’, ‘There is no significant relationship’, respectively)

We investigate how bidirectional relationships between COVID-19 incidence and human mobility are spatially distributed and how those spatial distributions change over time (i.e., across different waves). Figure 5 demonstrates the spatial variations in the relationship between COVID-19 incidence and human mobility with different directions of their relationship for each period. During wave 1, COVID-19 hit urban areas harder than rural areas [29]. The areas where COVID-19 Granger-causes mobility (in orange) also include metropolitan areas such as New York City, Chicago, Atlanta, Minneapolis, and San Francisco. This suggests that increased incidence of COVID-19 in large and dense metropolitan areas forced people reduce their mobility more substantially than in suburban and low-density urban areas. In contrast, wave 2 is when reopening policies started to be implemented, and many metropolitan areas of the South and Southwest experienced a surge. Some of those areas including Houston, Charlotte, and Las Vegas have a significant relationship that COVID-19 cases Granger-cause human mobility as shown in orange in Fig. 5. However, in this period, COVID-19 virus was not limited to metropolitan areas and had been widely spread to suburban and some rural areas as well [28]. As a result, the number of counties with significant relationships between COVID-19 cases and mobility in metropolitan areas became smaller. For example, some cities such as Chicago, Kansas City, and Indianapolis which had significant relationships in wave 1 are no longer significant in wave 2. Wave 3 is when the COVID-19 pandemic is the most severe with a peak of daily cases and deaths over the country. Like in wave 2, the number of counties where COVID-19 Granger-causes mobility keeps getting smaller in wave 3. Interestingly, on the other hand, the number of counties where mobility Granger-causes COVID-19 cases has increased. Indeed, in some areas such as Kansas City and Indianapolis where COVID-19 Granger-causes mobility in wave 1, the direction becomes the opposite in wave 3. Spatial variations during this period may be explained by the fact that the lift of lockdown policy was implemented by different states without a national mandate [8].

To further investigate if counties that have significant Granger causality are spatially clustered, we employed univariate local joint count statistics developed by Anselin and Li [30]. This is a local indicator of spatial association which is appropriate when a variable of interest is binary. This method allows us to statistically test if a county with a specific type of significant Granger causality result is surrounded by counties with the same significant Granger causality result than would be expected under conditions of spatial randomness. Here, we have two granger causality types: (1) COVID-19 incidence granger-causes mobility flows, and (2) Mobility flow granger-causes COVID-19 incidence. So, for each of three waves and for each of two granger causality types, we computed local join count statistics. Table 1 shows the number of counties that are significantly locally clustered with other counties with the same Granger causality type at the 0.05 level of significance. The numbers in parentheses indicate the total number of counties with significant Granger causality of each type. So, during wave 1 for example, there are 149 counties where COVID-19 Granger-causes mobility, and among them, 23 counties are locally clustered. Interestingly, these local clusters are mostly located near metropolitan areas including New York City (NY), Philadelphia (PA), Charlotte (NC), Chicago (IL), and Dallas (TX). The discovery of these clusters near these major metropolitan areas during different waves indicates that urban centers, with their higher population density and mobility, played a crucial role in the dynamics of COVID-19 spread and response.

**Table 1:** Number of counties that are locally clustered with other counties having the same Granger causality at the 0.05 level of significance

	Wave 1	Wave 2	Wave 3
COVID-19 → Mobility	23 (149)	3 (89)	5 (60)
Mobility → COVID-19	2 (44)	0 (9)	7 (57)

The numbers in parentheses indicate the total number of counties with significant Granger causality of each type

These findings reveal that counties with a particular type of Granger causality (either COVID-19 incidence Granger-causing mobility flows or mobility flows Granger-causing COVID-19 incidence) tend to be geographically clustered rather than randomly distributed. This clustering is observable in each of the three waves of the pandemic, for both types of Granger causality. The number of counties showing significant local clustering with similar Granger causality types suggests that the pandemic’s impact and the response in terms of mobility were not uniform across the U.S. but concentrated in specific regions.

### Assessing similarity between time series of COVID-19 incidence and human mobility

**Fig. 6:**
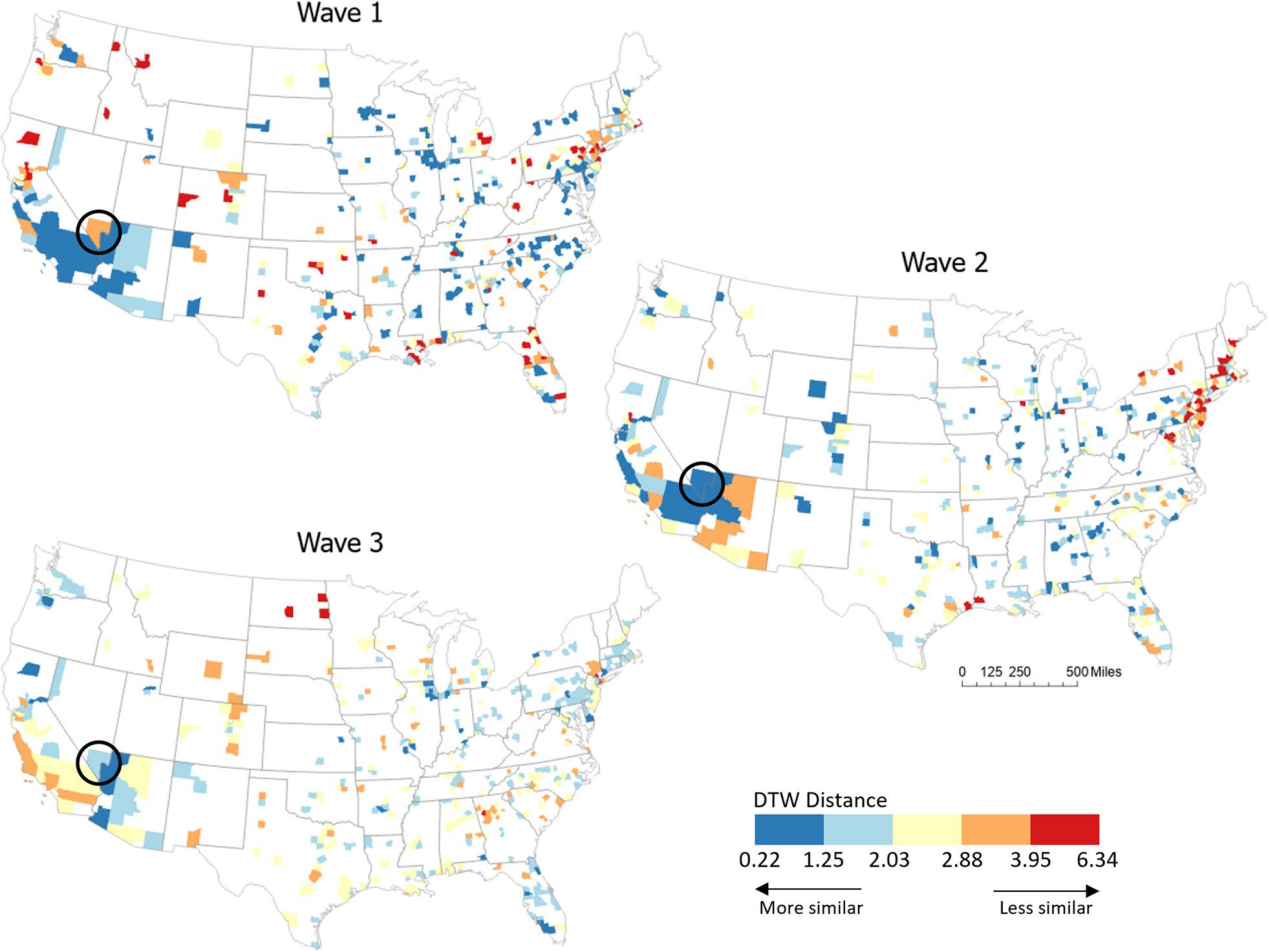
DTW distances for mobility and COVID-19 incidence in study areas. The value becomes smaller as two time series have more similar trends. Counties with shorter distances (in blue color hue) have more similar trends

The results from the Granger causality analysis identifies the existence of statistically significant associations between the two time series trends. However, the magnitude of association among the trends is unknown. We computed DTW distances between the standardized series of COVID-19 cases and mobility flows for each county and each wave to compare the similarity between the two trends across different geographies and throughout the different waves. Figure 6 highlights geographic variations in DTW distances over three time periods. The DTW distances are classified into five classes using Jenks’ natural breaks. To fairly compare different waves, we first defined classes based on all DTW distance values over three waves and then apply the same classification to all of the three waves. Overall, the degree of similarity is the highest in wave 1, and while it is gets gradually smaller in waves 2 and 3. This implies that the relationship between COVID-19 cases and mobility was strongest in wave 1, but other factors began to affect COVID-19 cases as the pandemic continued, which also corresponds with the results of the Granger causality test. Also, more importantly, the results from DTW demonstrate that even though there are statistically significant relationships between COVID-19 cases and mobility flows consistently over periods, the degree of such relationships may vary across space and time. For example, in Las Vegas, COVID-19 Granger-caused mobility flows in all three waves (Fig. 5); however, the DTW distance of that area was the largest in wave 2 and the smallest in wave 1 (circles in Fig. 6), which shows that the degree of their correlations changed over different waves.

To better understand the changes in the degree of similarity between COVID-19 case and mobility trends, we further investigated how DTW distances varied over time and whether the trends in cases and mobility were positively or negatively correlated (Table 2). We could confirm that the similarity between the two trends of COVID-19 cases and mobility flows became weaker, indicated by increasing DTW distances. More interestingly, however, the DTW distances showed notable differences based on the nature of the association (positive or negative) between the two trends, as determined by Pearson’s correlation coefficient. Specifically, when there was a positive correlation between COVID-19 incidence and mobility trends (i.e., both trends moved in the same direction), we found that the similarity in their patterns weakened. In contrast, a stronger similarity was observed when the trends were negatively associated (i.e., moving in opposite directions). Despite these variations, the overarching trend points to a general decline in the similarity between COVID-19 case numbers and mobility patterns. This finding suggests that the dynamics of the pandemic and public response, as reflected in mobility changes, have evolved in complexity. Initially, more direct relationships might have existed, but over time, factors such as changes in public behavior, and policy interventions could have influenced these patterns.

**Table 2:** Descriptive statistics of DTW distances between two trends of COVID-19 incidence and mobility flows

	Mean	Min	25%	50%	75%	Max
Wave 1						
Total	2.050	0.225	0.927	1.772	2.938	6.067
Positive association	1.088	0.225	0.673	0.983	1.388	2.644
Negative association	3.132	0.708	2.421	3.033	3.890	6.067
Wave 2						
Total	2.162	0.585	1.353	1.970	2.614	6.345
Positive association	1.557	0.585	1.033	1.431	1.931	4.708
Negative association	2.606	0.846	1.816	2.412	3.084	6.345
Wave 3						
Total	2.172	0.813	1.600	2.043	2.648	4.287
Positive association	2.042	0.813	1.398	1.907	2.484	3.853
Negative association	2.207	0.839	1.627	2.099	2.668	4.287

## Discussion and conclusions

During the COVID-19 pandemic, many countries have implemented non-pharmaceutical interventions (NPIs) that restrict human mobility such as stay-at-home orders to curtail the spread of the disease. To enhance the effectiveness of such interventions, it is crucial to understand the association between human mobility and COVID-19 incidence. Previous studies have indicated a positive correlation between COVID-19 incidence and human mobility, although this correlation has demonstrated variations across geographic regions and different phases of the pandemic. While it is recognized that COVID-19 incidence and human mobility can mutually influence each other, the intricacies of these bidirectional relationships remain incompletely understood. Furthermore, these bidirectional relationships may also exhibit variability across geographic regions and evolve over time, particularly as the pandemic persists with new waves and strains of the virus.

Our study presents an exploratory spatial time series analysis workflow designed to examine the spatial and temporal variations in bidirectional relationships between COVID-19 incidence and human mobility. Overall, our findings indicate that COVID-19 incidence had a more significant impact on mobility compared to the influence of mobility on COVID-19. The correlation between these variables was strongest during the initial wave of the pandemic and gradually weakened over time, primarily due to the implementation of NPIs in earlier waves. By utilizing a typology of change patterns, we identified shifts in the direction and significance of the relationship between COVID-19 incidence and mobility across different waves for specific counties. These shifts can be attributed to changes in disease control measures, risk perception, and the evolving behaviors of individuals, which vary both spatially and temporally. Furthermore, our workflow includes the application of dynamic time warping (DTW) to quantify the degree of similarity between COVID-19 cases and mobility flows by comparing their time series trends. Through visualizing standardized DTW distances, we provide insights into the spatial distribution of similar and dissimilar trends in disease incidence and mobility.

This study, however, has some limitations which need further investigation in future studies. In our analysis, we have primarily focused on examining the relationships between mobility flows and COVID-19 incidence across larger temporal scales defined by the pandemic waves. This approach aligns with our goal to capture broader trends and associations. We acknowledge that employing smaller temporal scales, such as a moving window of 2–3 weeks, could potentially uncover more detailed relationships within each defined period. For instance, the increasing and then decreasing pattern in case counts observed during each wave used in this study might be more distinctly segmented and analyzed by dividing this period into smaller sub-periods. Such a granular approach could reveal multiple occurrences and types of the “Granger-causing” relationships, offering a more detailed understanding of the interplay between mobility and infection rates. While acknowledging the significance and potential insights of such fine-scaled temporal analysis, it extends beyond the scope of our current study, which is designed to explore overarching trends across broader pandemic waves. This limitation, however, presents a promising direction for future research. Investigating the pandemic dynamics at a finer temporal resolution could provide valuable insights into the rapidly evolving nature of the pandemic and its interaction with human mobility patterns, thereby contributing to a more comprehensive understanding of pandemic behaviors and policy responses.

Another limitation is that we only consider human mobility flows as a factor that may affect COVID-19 or be affected by COVID-19. However, we acknowledge that many different factors, beyond mobility alone, play integral roles in understanding the dynamics of the pandemic. These factors, such as means of transport, availability to work from home, fear of infection, and the culture of communities, can have both direct and indirect influences on disease transmission. For example, the means of transport is a vital aspect often intertwined with mobility patterns. Variations in public transportation usage, personal vehicle use, and the availability of safe transport options can significantly affect not only the movement of individuals but also the spread of infectious disease. Additionally, the emotional and psychological aspect of fear related to infection can be another factor. Understanding how fear of infection influences mobility choices, social interactions, and adherence to preventive measures will help us better understand mobility behaviors and their association with disease incidence. Incorporating these additional factors beyond mobility into our analysis represents a promising direction for future research. It will allow us to gain more comprehensive understanding of the dynamics between disease incidence and human behaviors.

Finally, it's important to acknowledge certain aspects of data uncertainty and representativeness that were not explicitly addressed in this study. Our analysis utilizes daily COVID-19 case numbers and mobility flows, which are derived from mobile phone location data. While these datasets are invaluable for understanding the pandemic's dynamics and people's movement patterns, they inherently carry some level of uncertainty. This uncertainty stems from factors such as reporting delays, variations in testing rates for COVID-19, and the representativeness of mobile phone data for the entire population. Such uncertainties might have some impact on our findings, particularly when exploring the spatial and temporal variations in the relationship between COVID-19 and mobility trends. For example, the fact that not every person has a mobile phone and the SafeGraph mobile phone data is known to use a 10% sample of all cell phone providers in the U.S. obviously creates some bias. Although a recent study examined that the SafeGraph mobility data have relatively consistent sampling rate and highly correlated with the census population both in urban and rural areas at the county level [31], these data need to be analyzed and interpreted with caution. Moreover, regarding temporal variations, COVID-19 cases may be underreported in some periods, and different testing practices in different areas and time periods may also distort the actual trends of the disease. During wave 1, particularly, it was more difficult for the public to get a COVID-19 test compared to other phases [28]. The undercounting of COVID-19 cases can lower the data quality and hinder accurate analysis in COVID-19 research [32]. In future research, the COVID-19 data can be validated against external benchmarks, such as excess mortality estimates, which capture the number of deaths above the expected baseline. We can evaluate whether the reported COVID-19 data aligns with the overall increase in mortality during the pandemic. Incorporating validation against excess mortality estimates or other external benchmarks in future studies will help us address the uncertainties in COVID-19 case report data to ensure more precise correlations between COVID-19 incidence and human mobility.

Despite the limitations, our study contributes to a better understanding of the spatial and temporal dynamics of disease incidence in relation to human spatial interactions. This understanding is crucial as it helps reveal confounding factors and processes that influence disease dynamics. Additionally, our findings show evidence on how the outcomes of healthcare policies may vary at the city or county level, which provides a wide array of possibilities for location-based interventions to control the spread of COVID-19. Here, we highlight just three examples. First, NPIs such as lockdowns and travel restrictions could be dynamically adapted to location conditions. For instance, regions with a strong correlation between high COVID-19 incidence and increased mobility might benefit more from stricter mobility controls. Conversely, areas with lesser correlation could consider more nuanced restrictions, balancing disease control and socioeconomic activities. Second, the identification of areas where COVID-19 incidence significantly impacts mobility can help in directing public health messaging, and resources. In regions where the study shows that COVID-19 incidence didn't significantly deter mobility, there might be a need for more aggressive public awareness campaigns about the risks of mobility during high transmission periods. Third, by understanding the spatial and temporal trends of COVID-19 spread in relation to mobility, health authorities can allocate medical resources, testing facilities, and vaccination campaigns more effectively. Regions identified as high mobility despite high COVID-19 rates might require increased healthcare capacity or targeted vaccination drives.

Furthermore, while our study focuses on COVID-19 incidence and human mobility, the proposed methodology is generic and can be applied to explore time series associations between various spatiotemporal phenomena. For example, it can be extended to investigate the interactions between human mobility flows or contact tracing data and the evolution of other infectious diseases such as Influenza, which exhibit spatial and temporal variations.

## Data Availability

The county-level COVID-19 incidence datasets in the U.S. are available at COVID Act Now (https://covidactnow.org).

## Appendix: The distribution of the counties with Granger-causality across eight scenarios

We conducted a further investigation to show the distribution of the eight types of scenarios more clearly. To do so, for each county and for each wave, we performed additional analysis of (1) Pearson correlation to examine if two trends of COVID-19 and mobility have a positive or negative association and (2) a simple linear regression to decide if each of COVID-19 and mobility trends is increasing or decreasing. The Table 3 below shows the distribution of 272 counties that have at least one significant Granger-causality over three waves. This table illustrates the number of counties for each of eight scenarios. This gives us more detailed information about the relationship between COVID-19 and mobility trends. When COVID-19 trend Granger-causes mobility trend, they are more likely to have positive correlation where both trends are increasing in wave 1 and wave 2, but in wave 3, the most common scenario is that those two trends have a negative correlation where COVID-19 cases are increasing, and mobility flows are decreasing. On the other hand, when mobility trend Granger-causes COVID-19 trend, those two trends are more likely to have negative associations in waves 1 and 3, but positive associations are more common in wave 2. More specifically, in wave 2, positive association where both COVID-19 and mobility trends are increasing is the most common scenario.

**Table 3:** Distribution of the counties with Granger-causality across eight scenarios

		Wave 1	Wave 2	Wave 3
1	COVID-19 → mobility, positive correlation	0	0	10
	(decreasing COVID-19, decreasing mobility)			
2	COVID-19 → mobility, positive correlation	90	58	2
	(increasing COVID-19, increasing mobility)			
3	COVID-19 → mobility, negative correlation	59	15	0
	(decreasing COVID-19, increasing mobility)			
4	COVID-19 → mobility, negative correlation	0	16	48
	(increasing COVID-19, decreasing mobility)			
5	mobility → COVID-19, positive correlation	0	0	15
	(decreasing COVID-19, decreasing mobility)			
6	mobility → COVID-19, positive correlation	18	6	2
	(increasing COVID-19, increasing mobility)			
7	mobility → COVID-19, negative correlation	26	2	1
	(decreasing COVID-19, increasing mobility)			
8	mobility → COVID-19, negative correlation	0	1	39
	(increasing COVID-19, decreasing mobility)			
